# Accuracy of heart rate variability estimated with reflective wrist-PPG in elderly vascular patients

**DOI:** 10.1038/s41598-021-87489-0

**Published:** 2021-04-14

**Authors:** Christoph Hoog Antink, Yen Mai, Mikko Peltokangas, Steffen Leonhardt, Niku Oksala, Antti Vehkaoja

**Affiliations:** 1 Biomedical Engineering, KIS*MED, TU Darmstadt, Darmstadt, Germany; 2 Medical Information Technology, RWTH Aachen University, Aachen, Germany; 3 Faculty of Medicine and Health Technology, Tampere University, Tampere, Finland; 4 Finnish Cardiovascular Research Center, Tampere, Finland; 5 Center for Vascular Surgery and Interventional Radiology, Tampere University Hospital, Tampere, Finland; 6 PulseOn Oy, Espoo, Finland

**Keywords:** Predictive markers, Biomedical engineering, Optical sensors, Data processing

## Abstract

Optical heart rate monitoring (OHR) with reflective wrist photoplethysmography is a technique mainly used in the wellness application domain for monitoring heart rate levels during exercise. In the absence of motion, OHR technique is also able to estimate individual beat-to-beat intervals relatively well and can therefore also be used, for example, in monitoring of cardiac arrhythmias, stress, or sleep quality through heart rate variability (HRV) analysis. HRV analysis has also potential in monitoring the recovery of patients, e.g. after a medical intervention. However, in order to detect subtle changes, the calculated HRV parameters should be sufficiently accurate and very few studies exist that asses the accuracy of OHR derived HRV in non-healthy subjects. In this paper, we present a method to estimate beat-to-beat-intervals (BBIs) from reflective wrist PPG signal and evaluated the accuracy of the proposed method in estimating BBIs in a cross-sectional study with 29 hospitalized patients (mean age 70.6 years) in 24-h recordings performed after peripheral vascular surgery or endovascular interventions. Finally, we evaluate the accuracy of more than 30 commonly used HRV parameters and find that the accuracy of certain metrics, for example SDNN and triangular index, shown in the literature to be associated with the deterioration of the status of the patients during recovery from surgical intervention, could be adequate for patient monitoring. On the other hand, the parameters more affected by the high-frequency content of the HRV and especially the LF/HF-ratio should be used with caution.

## Introduction

Unobtrusive continuous monitoring and automatic analysis of physiological variables is an emerging area that has the potential to improve the effectiveness of healthcare delivery by providing early indications in the changes of the patients’ status, whether being treated in a hospital or staying at home. However, in order to be usable in practice, the data used by the automatic analysis algorithms needs to be reliable and accurate.

Reflective photoplethysmography (PPG) measured with a wrist-worn device, also called optical heart rate (OHR) monitoring, is a technique traditionally used mainly in the wellness application domain for monitoring heart rate level during exercise. In the absence of motion, the OHR technique is also able to estimate individual beat-to-beat intervals (BBIs) relatively accurately and has therefore recently emerged as an unobtrusive method for detecting cardiac arrhythmias^1–3^. Besides arrhythmias, the performance of wrist-worn OHR monitoring has also been studied, for example, in the assessment of psychological stress^4^ and in sleep staging through heart rate variability (HRV) and movement analysis in healthy subjects^5,6^. Studies evaluating the performance in beat-to-beat heart rate monitoring and accuracy of HRV parameters have usually been performed in controlled situations during selected activities or at rest as in^7,8^ or as reviewed in^9^. Further, studies evaluating the applicability of wrist-worn OHR technology in estimating HRV in hospitalised patients have been scantly reported.

A recent study reported poor performance of HRV estimation with commercial wrist OHR device in uncontrolled conditions^10^ highlighting the need for improvements in the measurement technology or signal analysis methods. The most significant limitation of OHR technology is its high sensitivity to movement artefacts, which poses challenges for the signal processing algorithms to choose only those heartbeats or heartbeat intervals that are not affected by movements. In addition, factors such as poor superficial blood perfusion and skin color affect the quality of the obtained signal and consequently, the accuracy of beat-to-beat heart rate^11,12^.

The predictive value of HRV parameters measured through gold standard electrocardiography (ECG) in identifying patients at risk of post-surgical complications has been studied in various patient groups such as in hip fracture^13^, digestive surgery^14^, abdominal aortic surgery^15^, and cardiac surgery patients. In cardiac patients, a popular topic has also been the evaluation of the relation of long-term mortality and HRV after myocardial infarction. A number of studies focusing on cardiac patients and HRV have been aggregated in the reviews by Nenna et al.^16^ and Huikuri and Stein^17^. HRV analysis has also been proposed for early detection of infections related to communicable diseases^18^, development of septic shock^19^, and also for several other purposes related to anesthesia and intensive care^20^.

Large number of HRV parameters have been found in the aforementioned studies to indicate a high risk of perioperative complications and post-surgery mortality. Short descriptions of these parameters and their abbreviations are found in Table 1. Ernst et al. found significant association with decreased RMSSD and total power of pre-operatively measured HRV and increased probability of post-operative complications as well as decreased VLF power and HF/LF-ratio and post-operative infections in hip fracture patients^13^. They, however, did not find association between pre-operative SDNN and post-operative complications. On the other hand, SDNN and HRV triangular index measured on post-operative day 1 were found to be statistically significantly lower in digestive surgery patients developing post-operative complications^14^. Both Nenna et al. as well as Huikuri and Stein identified scaling exponent $\alpha _1$ of detrended fluctuation analysis as a non-linear HRV parameter with high prognostic value in predicting long-term cardiac mortality^16,17^. This parameter was also found to be the best predictor of complicated recovery after coronary artery bypass grafting^21^. In the studies that have also evaluated the heart rate level as a potential indicator, increased post-operative heart rate has been found to predict or be associated with post-operative complications.

In all studies presented above, HRV analysis for monitoring of post-surgery patients and detecting complications has been performed using ECG. However, in order to be suitable for continuous monitoring for the duration of several days, the monitoring method should be as unobtrusive as possible. ECG electrodes are usually attached to the body with adhesives and may cause medical adhesive related skin injuries^22,23^, especially if worn for long periods of time, and are thus not a suitable approach.

The OHR technology would provide a convenient and unobtrusive solution for the task. However, typically the absolute amount of heart rate variation is decreased in aged and fragile patients^24^. As these patients exhibit the highest risk of developing complications, even better accuracy is needed from the measurement method in order to obtain adequate relative accuracy for the HRV parameters of interest. Inaccuracies in estimated heartbeat intervals also affect different HRV parameters differently^25^. Therefore, it is an interesting question whether those HRV parameters that have earlier been found to predict the development of post-treatment complications, can be estimated with adequate accuracy with OHR technology. We hypothesize that not all commonly used HRV parameters can be estimated with the same accuracy. We further hypothesize that some parameters may exhibit error levels small enough to render them possible candidates for future studies.

We performed 24-h monitoring with 29 subjects who had undergone vascular surgery or endovascular treatment. Typically, these patients have several comorbidities such as diabetes, hypertension, dyslipidemia, coronary artery disease, cerebrovascular disease and may have reduced cardiac function. For all patients, long-term recordings (mean 17.72 h, range 4.64–22.96 h) including day- and nighttime were acquired. The accuracy of a large set of HRV parameters was evaluated based on 5-min segments of BBIs estimated from reflective wrist PPG and reference ECG data.

## Methods

### Heartbeat interval and data quality estimation algorithm

For BBI estimation, we used continuous local interval estimation (CLIE) algorithm^26^ augmented with an iterative estimation approach (adaptive prior)^27,28^. The algorithm was originally developed for BBI estimation from ballistocardiography (BCG) data, which is often noisy and exhibits changes in morphology, rendering standard peak-detection strategies suboptimal. In a previous study, we have shown that the methodology is suitable for accurate, unbiased estimation of BBI intervals from clean (finger clip) PPG^29^: In short, intervals are estimated based on self-similarity of the underlying signal. For this, three estimators, namely short-term autocorrelation, maximum amplitude pairs, and mean absolute differences are fused. Estimation is performed iteratively, with the first iteration resulting in a prior signal that is used in the second pass. For details, the interested reader is referred to^28^.

**Figure 1:**
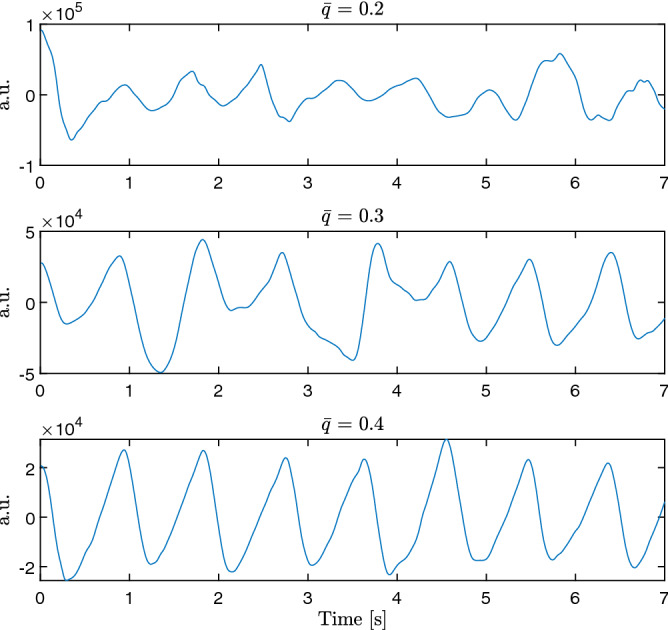
Three excerpts of the PPG-signal with varying levels of mean quality ${\bar{q}}$.

As in the original approach, in addition to the estimated interval, a quality metric *q* is reported for each estimated interval that quantifies the level of self-similarity detected. In Fig. 1, three signal excerpts are shown: while the first row presents an excerpt of a signal with a mean quality of 0.2, the second row shows an excerpt with ${\bar{q}} = 0.3$, and the last with ${\bar{q}} = 0.4$.

If a technology is used to detect slow changes in health status, it is often less critical to get continuous estimations, but more important that the accuracy of those is sufficient for the given task. Thus, a common approach is to exclude data based on a quality metric, thereby creating a tradeoff between the so-called (temporal) coverage and the error. In the particular case of HRV estimation, measurement protocols usually require the subject to be as calm as possible. As motion artifacts are the main source of error, it is particularly advantageous to exclude segments with low quality in such a scenario. In this work, four different quality metrics were used. First, we used a fixed threshold of $q_{\mathrm{th}}=0.3$ to only accept intervals with $q > q_{\mathrm{th}}$ in the subsequent analysis. In addition, for each 5-min window, the following rules were applied:

- Only if the median quality $q_{\mathrm{med}}$ of the window is above a threshold value $q_{\mathrm{th, med}}$, the window is accepted: $q_{\mathrm{med}} > q_{\mathrm{th, med}}$
- Only if the quality inside the window exhibits small variability (i.e. a small relative standard deviation), the window is accepted: $\mathrm{SD}(q) / {\overline{q}} < q_{\mathrm{th, var}}$
- Only if the ratio of accepted intervals inside the window $N_{\mathrm{OK}}$ relative to the estimated mean interval $\bar{\mathrm{BBI}}$ is large enough, the window is accepted: $N_{\mathrm{OK}} / \bar{\mathrm{BBI}} > R_{\mathrm{th, OK}}$

**Figure 2:**
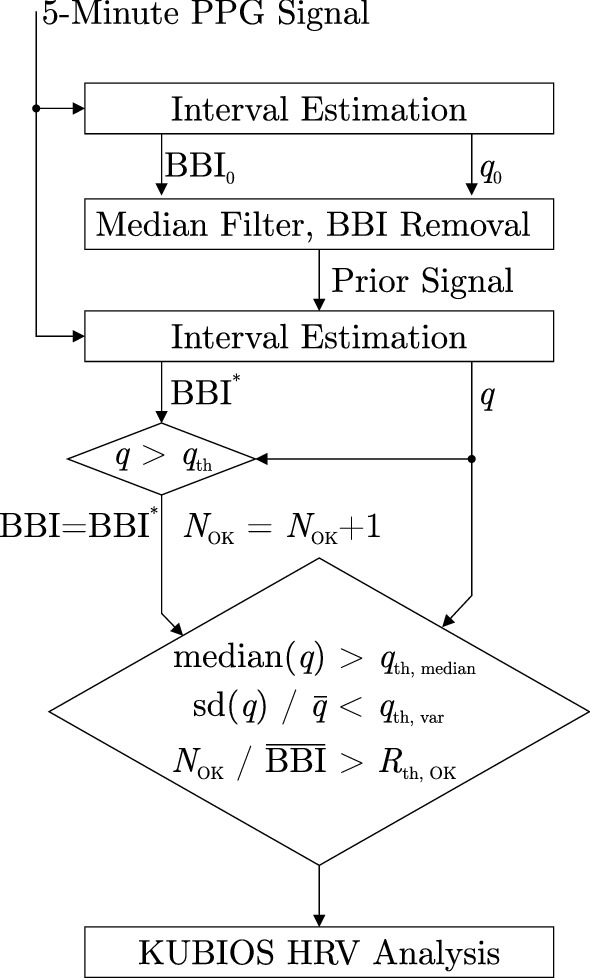
Overall flow of the BBI estimation approach. The BBI-time series is estimated using an iterative approach^28^. Only if three criteria (sufficient median quality $q_{\mathrm{th, median}}$, low variation in quality $q_{\mathrm{th, var}}$, ratio of intervals estimated $R_{\mathrm{th, OK}}$) are met, the 5-min window is analyzed using the Kubios HRV analysis software.

The threshold values can be chosen by the user based on priorities of the application i.e. large data coverage or accuracy (as will be shown in the results section, Figs. 4 and 5). The overall process is visualized in Fig. 2.

### HRV parameters

To calculate HRV parameters from both the reference ECG and the PPG-derived BBIs, the analysis software “Kubios HRV premium” by Kubios Oy, Finland, is used. Kubios allows the calculation of more than 30 HRV parameters^30^ and has beed used in more than 4500 scientific publications according to the manufacturer. In this study, the parameters presented in Table 1 were evaluated.

**Table 1:** HRV parameters calculated using the “Kubios HRV Premium” software.

HRV parameter description	Abbreviation
Energy expenditure	EE
Training impulse	TRIMP
Stress index	SI
Mean of normal-to-normal (NN) intervals	Mean NN
Standard deviation of NN intervals	SDNN
Mean heart rate	Mean HR
Standard deviation of heart rate	SDHR
Minimum heart rate	Min. HR
Maximum heart rate	Max. HR
Root-mean-square of successive differences	RMSSD
Number of interval differences $> 50\,\hbox {ms}$	NN50
Percentage of interval differences $> 50\,\hbox {ms}$	pNN50
Triangular index	Tri. Indx
Triangular interpolation of NN intervals	TINN
Poincaré $\hbox {SD}_1$	PC $\hbox {SD}_1$
Poincaré $\hbox {SD}_2$	PC $\hbox {SD}_2$
Poincaré $\hbox {SD}_2$/$\hbox {SD}_1$	PC $\hbox {SD}_2$/$\hbox {SD}_1$
Approximate Entropy	ApEn
Sample entropy	SampEn
Scaling exponent $\alpha _1$ of detrended fluctuation analysis	DFA $\alpha _1$
Scaling exponent $\alpha _2$ of detrended fluctuation analysis	DFA $\alpha _2$
Very low frequency (VLF, 0–0.04 Hz) peak frequency	VLF PF
Low frequency (LF, 0.04–0.15 Hz) peak frequency	LF PF
High frequency (HF, 0.15–0.4 Hz) peak frequency	HF PF
VLF absolute power	VLF Abs.
LF absolute power	LF Abs.
HF absolute power	HF Abs.
Natural logarithm of VLF absolute power	VLF Log.
Natural logarithm of LF absolute power	LF Log.
Natural logarithm of HF absolute power	HF Log.
VLF relative power	VLF Rel.
LF relative power	LF Rel.
HF relative power	HF Rel.
LF normalized power	LF Norm.
HF normalized power	HF Norm.
LF power to HF power ratio	LF/HF
Parasympathetic nervous system index	PNS Indx
Sympathetic nervous system index	SNS Indx

In particular, the analysis includes several time-domain parameters (SDNN, SDHR, RMSSD), frequency-domain parameters associated with low-frequency components (LF Abs., LF Log.), frequency-domain parameters associated with high-frequency components (HF Abs., HF Log.), as well as relative (Poincaré $\hbox {SD}_2$/$\hbox {SD}_1$, LF/HF), statistical (NN50, pNN50), and nonlinear (ApEn, SampEn, PC $\hbox {SD}_{1,2}$, DFA $\alpha _{1,2}$) parameters. The 5-min analysis window was shifted in steps of 60 seconds and HRV parameter calculation repeated. HRV parameters estimated with Kubios from PPG-based BBI and corresponding ECG-based reference RR-interval windows were exported and comparisons were performed in MATLAB.

For each 5-min window of the data of each subject, a ground-truth HRV parameter and an estimated value exist. For evaluation, we performed per-subject analyses (Figs. 6 and 8) as well as combined gross analyses (all other figures and tables). In the per-subject analysis, error metrics are calculated for each subject individually. This implies that the relative error is calculated by normalization with the average of the ground truth of all windows of that subject. In the gross analysis, all data of all subjects are aggregated. Here, the relative metrics are based on the average ground truth values of all windows of all subjects. Thus, the gross analysis is biased towards subjects with more accepted datapoints. By comparing gross and per-subject analysis, information about inter-subject variability is obtained.

To provide information on the distribution of the error, several error metrics are used. For all metrics, the difference of estimation and ground truth ($\Delta =$ estimation − ground truth) of the respective HRV parameter forms the basis. The “Bias” is defined as the average of all $\Delta$, i.e. it indicates over- and under-estimation in absolute units. The relative bias “Bias (%)” is the bias normalized by the average of the ground truth. “P05” and “P95” are defined as the 5th and 95th percentile of $\Delta$ and give an indication of the spread of the error in absolute units. Similarly, the standard deviation of $\Delta$, “SD” is a metric for the spread of the error assuming a Gaussian distribution. The mean absolute error “MAE” is defined as the mean of the absolute values of $\Delta$, while the root mean square error “RMSE” is determined by calculating the root of the mean value after squaring all individual $\Delta$. As the RMSE is more sensitive to outliers, the comparison of MAE and RMSE gives information about their presence. The relative quantities “MAE (%)” and “RMSE (%)” are calculated analogously to the relative bias, with the selection of the normalization depending on per-subject or gross analysis as described above. Finally, we analyze the “Relative Error”, which is $\Delta$ divided by the mean of the ground truth. While “Bias (%)” is equivalent to the mean of “Relative Error”, we additionally provide median, 25th, and 75th percentile to obtain further information of the spread of the error in relative terms (Fig. 7).

### Evaluation data

**Figure 3:**
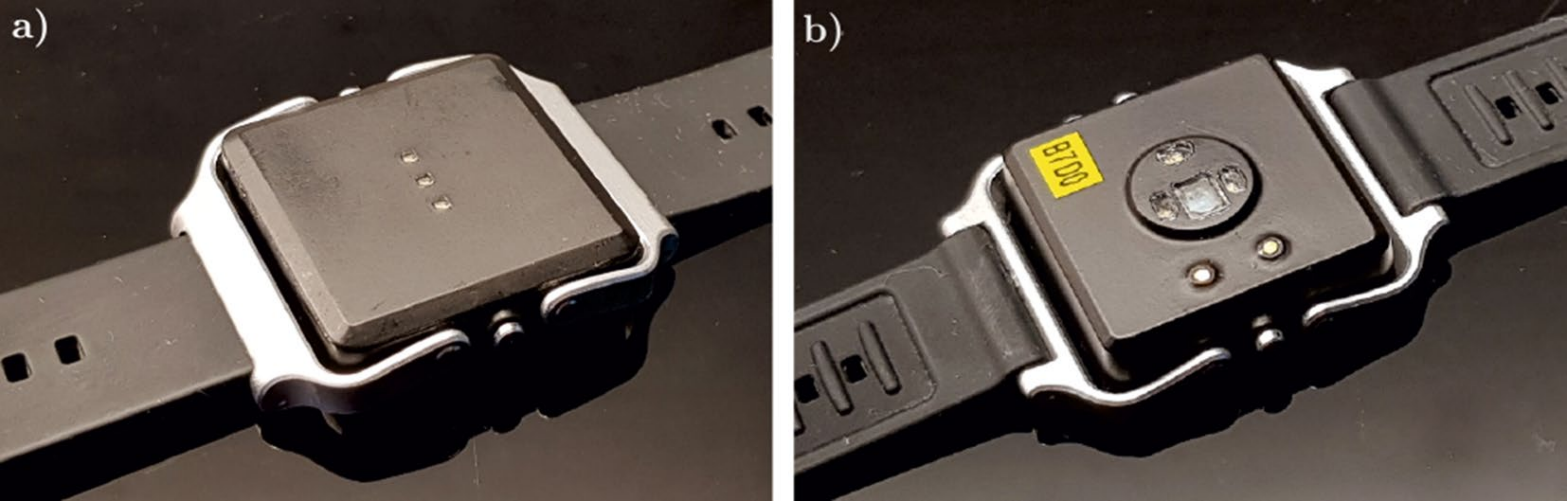
Optical heart rate monitoring device (prototype) used in the study. (**a**) top view with status LEDs, (**b**) bottom view (facing the measurement area).

The patient recordings were performed at the vascular surgery ward of Tampere University Hospital between April and October 2018. Inclusion criteria for the study were at least 50 years of age and admission to peripheral arterial bypass operation, endarterectomy, aortic surgery, or carotid surgery. Patient with cardiac pacemaker were excluded from the study. The study was a descriptive pilot study for gaining initial knowledge about the performance and suitability of new sensor technology for patient monitoring and 30 successful patient recordings was determined as a suitable sample size. Altogether 36 patients were recruited for the study but seven subjects had to be discarded due to technical problems or due to short duration of the recording. In four cases the problem was with the reference device (poor quality ECG), in one case with the study device (recording had not started), and in two cases the patient was admitted to re-operation shortly after the beginning of the recording. Thus, the data of 29 postoperative vascular patients were included in the analysis. The subjects were monitored for approximately 24 h with a wrist-worn OHR prototype device manufactured by PulseOn Oy, Finland, Fig. 3. Long-term recordings were obtained to eliminate bias potentially arising from, for example, monitoring only sleeping subjects.

The PulseOn wrist device uses green color LEDs with peak wavelength of 573 nm and 25 Hz sampling rate. Before interval estimation, the PPG data were upsampled to 200 Hz using linear interpolation and bandpass-filtered using a 2nd-order Butterworth bandpass-filter with a passband of 0.5 to 15 Hz.

The reference ECG was recorded with a Faros 360 five-lead Holter monitor manufactured by Bittium Biosignals using 1 kHz sampling frequency. Ambu Blue sensor L-00-S electrodes were used for the ECG recording. The average age of the subjects was 70.6 years (SD: 8.5 years, range 50–87 years). Seven of the subjects were female. The subjects had undergone different vascular and endovascular procedures such as lower limb percutaneous transluminal angioplasty and/or stenting, abdominal aortic aneurysm endovascular repair, carotid or femoral artery endarterectomy, or femoropopliteal bypass surgery. Approval for this study was obtained from the Regional Ethical Committee of Pirkanmaa Hospital District (R17027). Informed consent was obtained from all subjects. The guidelines of the Declaration of Helsinki were followed in the study. The study was registered at ClinicalTrials.gov, identifier NCT03572751.

In this study, ECG and PPG were recorded with two independent, unconnected wearable devices. Devices that have independent system clocks and are not synchronized can exhibit drifts in sampling rate^31^. In long-term recordings, even small drifts can amount to large offsets, which would lead to the comparison of non-corresponding windows in this study. Thus, we adopted the same alignment process as in our previous work on BCG data^28^: The algorithm calculates a time-varying offset that minimizes the median BBI estimation error in a moving window with the size of 1500 beats, which corresponds to approximately 25 min. The offset-vector was additionally median filtered with the same filter size to remove outliers. Finally, an offset-vector is obtained that ensures that each window of the PPG data is compared to the matching ECG window.

## Results and discussion

**Figure 4:**
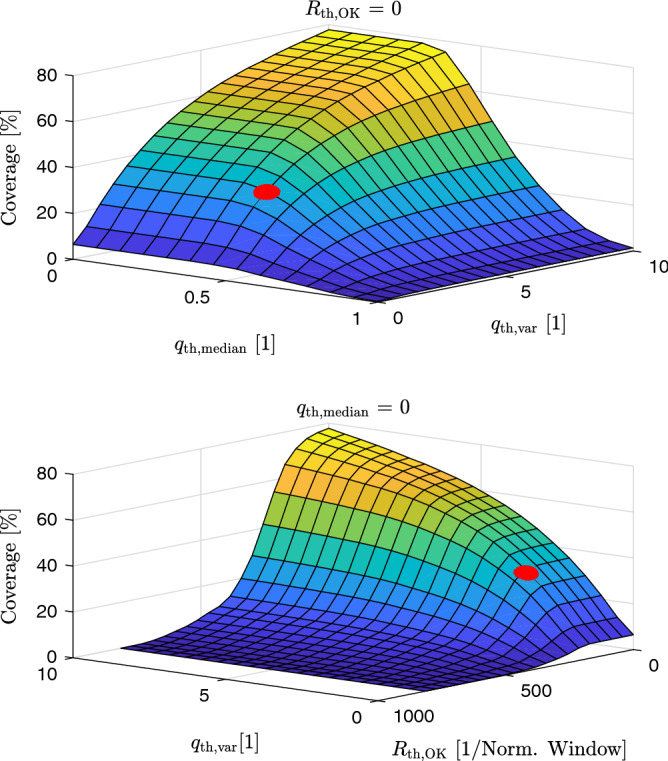
Influence of the threshold-parameters on the coverage. The red dot indicates the thresholds used in the final analysis.

**Figure 5:**
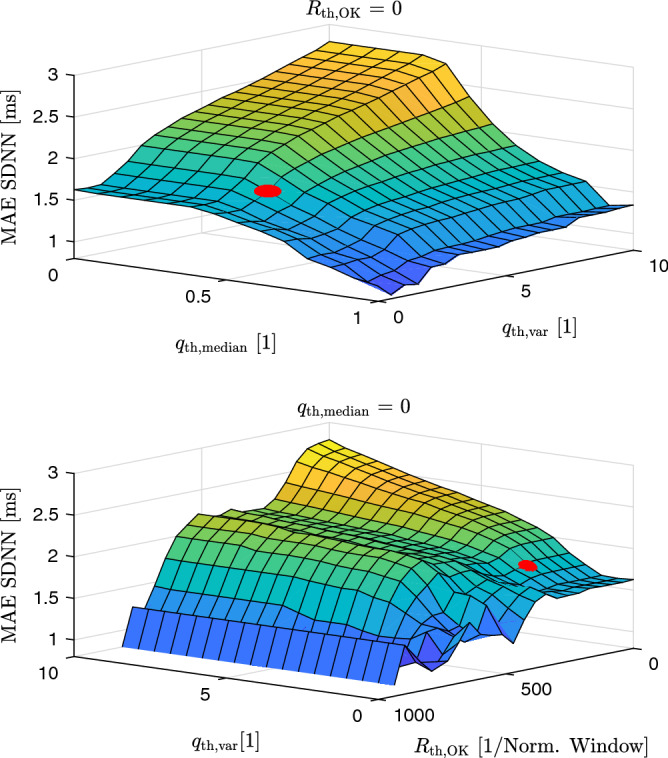
Influence of the threshold-parameters on the mean absolute error (MAE) of the SDNN parameter. The red dot indicates the thresholds used in the final analysis.

Figures 4 and 5 demonstrate the effect of the threshold-parameters on the coverage of accepted 5-min windows and the mean absolute error of the HRV parameter SDNN, respectively.

As expected, the coverage decreases monotonically with an increase in $R_{\mathrm{th, OK}}$, an increase in $q_{\mathrm{th, median}}$, and a decrease in $q_{\mathrm{th, var}}$ (Fig. 4). The same general tendency holds for the estimation error. Fig. 5 shows the mean absolute error for SDNN as one example. Note that the distribution of the coverage is the same for all HRV parameters, whereas we observed varying dependencies on the three thresholding parameters for the different error metrics and the different HRV parameters (not shown). In the following, a target coverage of 30% was arbitrarily set. Out of the several combinations of thresholds that would result in this target coverage, the following set was chosen:

- $$R_{\mathrm{th, OK}} = 171.22$$
- $$q_{\mathrm{th, median}} = 0.46$$
- $$q_{\mathrm{th, var}} = 2.11$$

This choice of thresholds is visualized with a red dot in Figs. 4 and 5. Note that while these parameters lead to an *average* coverage of 30 %, the coverage for individual subjects may vary. Further note that this choice is fixed for all following calculations and arbitrary in the sense that different combinations of thresholds would also lead to the same average coverage of 30% as can be inferred from Fig. 4. At the same time, it would lead to different error levels, as can be seen in Fig. 5. As a consequence, the arbitrarily chosen parameters mark the upper bounds of the errors of the individual HRV parameters.

**Figure 6:**
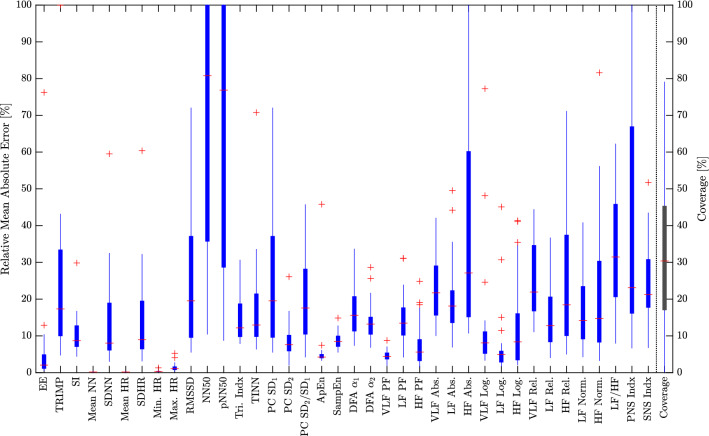
Per-Subject analysis of the relative mean absolute error, i.e. each data point in the bar graphs represents one of the 29 subjects. For better readability, the graph is clipped at 100 %. The last bar on the right shows the distribution of the HRV coverage.

Table 2 shows the numeric comparison of 38 different HRV parameters calculated for all accepted 5-min windows and aggregated for all 29 (out of the original 36) patients included into the analysis (i.e. so-called “gross analysis”, see Sect. 2.2). In Fig. 6, per-subject results are presented as boxplots, where each datapoint presents results for one individual subject. Note that the graph is clipped at 100 % for better readability.

**Table 2:** Accuracy of the estimated HRV parameters aggregated over all analyzed 5-min windows of all subjects.

Parameter		MAE	MAE (%)	RMSE	RMSE (%)	P05	P95	Bias	Bias (%)	SD
EE	[kcal/min]	0.0213	1.49	0.1540	7.33	− 0.0501	0.0293	0.0018	0.13	0.1540
TRIMP	[kTRIMP/min]	0.1852	6.55	1.3042	10.23	0.0000	0.2963	0.0986	3.49	1.3006
SI	[–]	2.2950	10.75	3.7738	16.29	− 7.9781	2.2682	− 1.4460	− 6.77	3.4860
Mean NN	[ms]	1.8748	0.20	4.0133	0.42	− 1.7276	7.0315	1.1709	0.12	3.8390
SDNN	[ms]	1.7143	8.94	2.8611	12.66	− 3.0127	3.7421	0.3891	2.03	2.8347
Mean HR	[BPM]	0.1259	0.20	0.2646	0.41	− 0.4574	0.1544	− 0.0645	− 0.10	0.2566
SDHR	[BPM]	0.1292	10.18	0.2756	18.93	− 0.2222	0.2816	0.0251	1.98	0.2745
Min. HR	[BPM]	0.1884	0.31	0.5274	0.85	− 0.1429	0.6322	0.0931	0.15	0.5192
Max. HR	[BPM]	0.8199	1.18	2.6971	3.83	− 3.3757	0.2343	− 0.7047	− 1.02	2.6036
RMSSD	[ms]	3.5506	17.15	4.5453	18.34	− 3.4138	8.3910	2.4160	11.67	3.8504
NN50	[beats]	4.5662	28.60	8.2695	23.95	− 10.0000	14.0000	1.5054	9.43	8.1319
pNN50	[%]	1.5457	28.00	2.7441	22.59	− 2.6778	5.1406	0.7025	12.73	2.6529
Tri. Indx	[–]	0.6585	12.19	0.8661	14.31	− 1.4889	1.2480	0.0289	0.54	0.8657
TINN	[ms]	11.7354	12.14	22.0286	19.15	− 23.0000	24.0000	1.7424	1.80	21.9612
PC $\hbox {SD}_1$	[ms]	2.5147	17.15	3.2191	18.33	− 2.4170	5.9416	1.7116	11.67	2.7266
PC $\hbox {SD}_2$	[ms]	1.5550	6.94	3.4167	12.79	− 4.1039	2.3250	− 0.4611	− 2.06	3.3857
PC $\hbox {SD}_2$/$\hbox {SD}_1$	[–]	0.3073	18.78	0.4471	25.68	− 0.9732	0.0776	− 0.2867	− 17.52	0.3430
ApEn	[–]	0.0496	4.47	0.0735	6.59	− 0.1008	0.0899	− 0.0060	− 0.54	0.0733
SampEn	[–]	0.1552	8.25	0.2013	10.63	− 0.2462	0.3681	0.0634	3.37	0.1910
DFA $\alpha _1$	[–]	0.1502	16.99	0.2044	21.83	− 0.4356	0.1098	− 0.1164	− 13.16	0.1680
DFA $\alpha _2$	[–]	0.0594	13.29	0.0860	17.86	− 0.1801	0.0303	− 0.0497	− 11.12	0.0702
VLF PF	[mHz]	1.5315	4.47	3.7685	10.87	− 6.6667	6.6667	0.0508	0.15	3.7684
LF PF	[mHz]	9.1981	15.40	20.6890	32.38	− 23.3333	40.0000	2.5509	4.27	20.5326
HF PF	[mHz]	16.7561	6.44	39.6176	14.86	− 36.6667	63.3333	3.3309	1.28	39.4802
VLF Abs.	[$\hbox {ms}^2$]	13.2536	19.35	36.9939	25.97	− 51.4332	5.0578	− 10.4884	− 15.31	35.4786
LF Abs.	[$\hbox {ms}^2$]	33.9061	15.82	208.0664	42.14	− 103.4176	31.8021	− 20.5115	− 9.57	207.0681
HF Abs.	[$\hbox {ms}^2$]	35.8349	18.55	63.0736	18.18	− 83.6440	68.3491	4.8635	2.52	62.8905
VLF Log.	[log]	0.2509	7.80	0.4562	12.85	− 0.6521	0.2926	− 0.1318	− 4.16	0.4368
LF Log.	[log]	0.2038	4.63	0.4208	9.07	− 0.4128	0.4864	0.0082	0.19	0.4208
HF Log.	[log]	0.3819	8.56	0.6216	13.35	− 0.3093	1.1854	0.2692	6.04	0.5603
VLF Rel.	[%]	3.4880	23.93	5.4798	29.68	− 12.0000	1.9409	− 2.8145	− 19.31	4.7021
LF Rel.	[%]	6.0661	14.95	8.8321	20.03	− 18.4170	7.4994	− 3.2157	− 7.92	8.2265
HF Rel.	[%]	8.2323	18.36	11.8936	23.76	− 6.6075	25.5536	6.0302	13.45	10.2524
LF Norm.	[n.u.]	8.1777	16.70	11.8355	22.05	− 25.3497	7.2359	− 5.6440	− 11.52	10.4039
HF Norm.	[n.u.]	8.1777	16.03	11.8355	21.31	− 7.2359	25.3497	5.6440	11.06	10.4039
LF/HF	[n.u.]	0.5818	35.95	1.1213	44.10	− 2.5853	0.2765	− 0.4846	− 29.95	1.0112
PNS Indx	[–]	0.1518	19.70	0.1935	20.00	− 0.1014	0.3836	0.1209	− 34.93	0.1512
SNS Indx	[–]	0.3924	20.50	0.6327	25.28	− 1.3293	0.3248	− 0.2798	− 16.47	0.5675
Mean	[%]		12.92		17.60				− 2.72	
Median	[%]		12.74		18.26				0.14	

Several observations can be made from the results. For one, the (relative) RMSE tends to be significantly higher than the (relative) MAE for many of the parameters, indicating the tendency for large outliers in the estimation results. Moreover, estimation quality varies greatly from patient to patient and strongly depends on the parameter. While mean, maximum, and minimum heart rate can be estimated with an average relative error below 1.2 %, other parameters show inferior results. In particular, the patient-wise relative error in NN50 and pNN50 has a 75th percentile at about 280 % (not shown in Fig. 6 due to clipping). For one, the nature of this parameter makes it relatively susceptible to outliers. For another, the population in this study group has extremely low NN50/pNN50 values close to zero. As can be seen in Table 2, the mean absolute error in pNN50 is only 1.55 percentage points (which would obviously still result in an infinite error for patients with a true pNN50 of 0 %).

**Figure 7:**
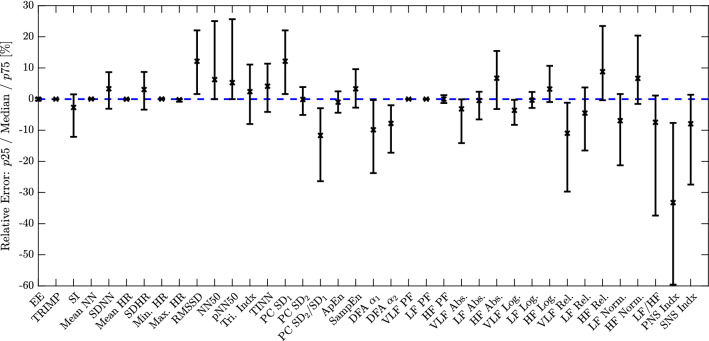
Aggregated gross analysis over all subjects in terms of relative error for all parameters. The crosses indicate the median value. The lower bar marks the 25th percentile, the upper bar the 75th percentile.

Figure 7 visualizes bias and spread of the error aggregated over all subjects and normalized by the overall mean of the ground truth in percent. The bias is given as median and the spread as 25th/75th percentiles. It can be seen that the time-domain estimations (SDNN, SDHR) and the nonlinear Poincaré SD2 have been estimated with only small biases. On the other hand, the components RMSSD and Poincaré SD1 exhibit a systematic overestimation in the range of 10 % with an interquartile range of approximately $\pm 10\,\%$. This tendency is largely a result of arbitrary/sporadic large beat-to-beat interval estimation errors, which tend to affect parameters containing differentiations in a more severe way. Note that the same systematic overestimation can also be seen in the frequency-domain parameters associated with high-frequency components.

In the frequency domain, both absolute as well as relative power are estimated with large biases and spreads. If comparison is performed in the logarithmic domain, however, biases/spreads are small for VLF, LF, and HF components ($-4\pm 5\,\%$, $0\pm 3\,\%$, $3\pm 7\,\%$). As a general tendency, we observe that high-frequency associated parameters HF Abs. and HF Log. as well as PC $\hbox {SD}_1$ and RMSSD are systematically overestimated. While these biases may be small in absolute numbers ($4.86\,\hbox {ms}^2$ and 0.27 log, 1.71 ms and 2.42 ms, respectively), they do lead to a comparatively large relative bias in the range of 10 % for our patient group. Consequently, parameters that analyze the ratio of LF to HF components (LF/HF, PC $\hbox {SD}_2$/$\hbox {SD}_1$) show severe systematic underestimation.

Note that the per-subject analysis (Fig. 6) calculates the relative error as “average of the error of subject *n* divided by average of the ground truth of subject *n*”. The aggregated gross analysis, on the other hand, calculates “average of the error of all windows of all subjects divided by average of the ground truth of all windows of all subjects” (Table 2). Interesting observation can be made comparing the two: First, strong inter-individual differences can be observed in Fig. 6 and the optical measurement clearly seems to be more suitable for certain individuals than for others. For the “best” 25 % of the subjects, the relative MAE of most of the HRV parameters is less than 10 %. Second, as the relative per-subject errors far exceed those of the gross analysis in Table 2, we can assume that estimation errors in subjects with very low HRV parameter values have a strong negative impact on the relative accuracy seen in Fig. 6.

**Figure 8:**
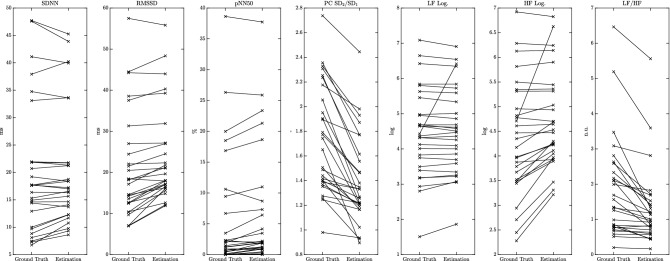
Comparison of ground truth and BBI-based estimation for seven selected HRV parameters. Each ‘x’ marks the mean over all windows where an estimation from PPG was available (Estimation) and the mean over all corresponding reference windows obtained from ECG (Ground Truth).

These assumptions can be supported further by Fig. 8. Here, each ‘x’ marks the mean over all windows where an estimation from PPG was available (‘Estimation’, right) and the mean over all corresponding reference windows obtained from ECG (‘Ground Truth’, left).

The graph shows data from 28 patients, as the coverage for one patient was zero (see also Fig. 6, rightmost column). Indeed, several patients exhibit a pNN50 of (close to) zero. Nevertheless, five patients show pNN50-values way above 10 % that can clearly be distinguished also in the PPG-based estimation. The same tendency holds for SDNN and LF Log. and, in parts, for RMSSD and HF Log., although for these parameters the aforementioned over-estimation of small values becomes obvious. Finally, neither LF/HF nor PC $\hbox {SD}_2$/$\hbox {SD}_1$ can be estimated with confidence due to strong and, more importantly, varying under-estimations as indicated by several crossing lines.

As can be seen in our HRV parameter estimation accuracy evaluation, from the aforementioned parameters, SDNN was estimated on average with 9 % and triangular index with 12 % relative MAE from the wrist device PPG signal. The relative MAE of both DFA $\alpha _1$ and RMSSD parameters were approximately 17 % and the absolute power of LF and HF components between 16 % and 19 %. The LF/HF ratio performed the worst with 36 % relative MAE. The relative MAE of ApEn parameter was only approximately 5 %, which is partially explained by the distribution of the parameter values, i.e. small deviation compared with the average. Further, for the parameters such as triangular index and approximate entropy that are showing low biases, the increase of the estimation window could improve the accuracy.

Previous studies have proposed that the changes in HRV parameters caused by postoperative complications and deterioration of the patient status can be seen on post-operative day 1, for example, in SDNN and triangular index^14^ as well as in DFA scaling exponent $\alpha _1$^21^. In^21^, Laitio et al. reported mean ± standard deviation of DFA $\alpha _1$ of $0.85\pm 0.17$ vs. $0.69\pm 0.18$ for two groups of patients, “ICU Stay $\le 48 \,\hbox {h}''$ and “ICU Stay $>48\,\hbox {h}''$, respectively. Based on the results obtained in the present study, it is uncertain whether or not these two groups could be separated using OHR: For DFA $\alpha _1$, we obtained an MAE of 0.150 and an RMSE of 0.204 across all data, which lies in the range of the differences between the two groups. On the other hand, Ushiyama et al. found mean ± standard deviation of SDNN for the “complicated” group being $48.7\pm 24.4\,\hbox {ms}$, whereas $71.2\pm 19.6\,\hbox {ms}$ was found for the “uncomplicated” group of digestive surgery patients, see^14^. Based on the observed accuracy for SDNN (1.71 ms MAE, 2.86 ms RMSE), we would argue that these groups could clearly be separated with the OHR technology. The same argument can be made for the Triangular Index ($13.3\pm 6.7$ “complicated” group, $19.9 \pm 6.5$ “uncomplicated” group^14^), for which we found an MAE of 0.659 and an RMSE of 0.866.

In^19^, the development of septic shock was most clearly predicted by RMSSD but also by absolute and normalized LF power as well as HF power and LF/HF-ratio. However, median values of (as well as differences between) both groups were extremely low: For example, comparing “No septic shock” with “Septic shock”, the median values of RMSSD for both groups were 3.8 ms and 7.3 ms, respectively^19^. It thus remains questionable if the accuracy obtained in the present study with OHR (3.55 ms MAE and 4.55 ms RMSE for RMSSD) would suffice for this application scenario. Approximate entropy has also been found to predict the onset of atrial fibrillation (AFib) after coronary artery bypass grafting^32^. The “control patients” exhibited mean ± standard deviation values of $1.04\pm 0.05$, whereas a decreased ApEn of $0.93\pm 0.05$ during 1 h preceding the AF onset was measured in^32^. Our observed error levels for ApEn of 0.0496 MAE/0.07354 RMSE indicate that ApEn measured with OHR could potentially be directly used for AFib prediction.

Although the presented study is, to the best of our knowledge, the most comprehensive one in terms of assessing the accuracy of HRV estimations obtained via OHR in hospitalized patients, all-encompassing statements about the most reliable parameters may not be possible. Nevertheless, we believe our findings in terms of parameter recommendations can be summarized as follows:

- The time-domain parameter SDNN can be estimated with a relative error/absolute error/relative bias of 9%/2 ms/2% and we thus recommend its use. We also recommend the Triangular Index with 12%/0.66/1%. The parameters RMSSD and pNN50 exhibit large relative errors in this cohort (17% and even 28%) and systematic biases in the range of 12%, but also low absolute estimation errors (4 ms and 2%). The visualization in Fig. 8 further suggests that patients with low and high RMSSD/pNN50 might very well be separated using OHR technology. Thus, we believe these parameters should be investigated further.
- In the frequency domain, the relative errors for LF Abs. and HF Abs. were 16% and 19%, while the corresponding values for LF Log. and HF Log. were only 5% and 9%. We thus recommend the use of the logarithmic quantities, although one needs to analyze whether they possess the same discriminative power as the absolute ones. Again, Fig. 8 suggests that separation of patients would be possible.
- In their current implementation, the relative quantities (LF/HF, PC $\hbox {SD}_2$/$\hbox {SD}_1$) show strong relative errors (36% and 19%) and biases (− 30% and − 18%) and cannot be recommended without improving their estimation.

Finally, the patient cohort consisted predominantly of white caucasian subjects. Thus, the generalisability of results has to be validated with a more diverse group. Although we do not expect a fundamentally different outcome in terms of parameter feasibility, overall estimation errors may be higher in subjects with darker skin tones.

## Conclusion

In conclusion, the accuracy of HRV parameters estimated from the PPG signal of wrist-worn OHR monitoring device varies significantly between parameters and subjects. The accuracy of certain parameters, for example SDNN and triangular index, shown in the literature to be associated with the deterioration of the status of the patients during recovery from surgical intervention, could be adequate for patient monitoring. On the other hand, the parameters more affected by the high-frequency content of the HRV and especially the LF/HF-ratio should be used with caution. It should also be emphasized that the proposed data analysis method tries to discard such segments of PPG signal that likely produce less reliable beat-to-beat interval data resulting the HRV parameters being obtained only for approximately 30% of the time. This may limit the usability of the approach in some applications. To further improve the applicability of wrist-worn OHR monitoring in patient surveillance through HRV, more robust methods for beat-to-beat interval estimation and especially methods for mitigating the effect of estimation uncertainty on the HRV parameter values, e.g. robust spectral estimation techniques, should be investigated.
